# Exploring the influence of food labels and advertisements on eating habits of children: a cross-sectional study from Punjab, India

**DOI:** 10.1186/s12889-023-15058-3

**Published:** 2023-02-11

**Authors:** Madhur Verma, Ramnika Aggarwal, Bhola Nath, Rakesh Kakkar

**Affiliations:** 1 Department of Community and Family Medicine, All India Institute of Medical Sciences, Bathinda, Punjab 151001 India; 2 Departmentof Community and Family Medicine, All India Institute of Medical Sciences, Raebarely, Uttar Pradesh 229405 India

**Keywords:** Childhood obesity, Front-of-pack labeling, Obesogenic environment, Healthy eating

## Abstract

**Background:**

Maintaining healthy eating habits among children is challenging due to industrial tactics. There is little research on the effect of nutritional labels and tv ads on the eating habits of children. So the primary aim of the study was to explore the noticeability of the food packaging labels by the children, the information retrieved from the food nutrition labels, and their role in increased frequency of eating out in addition to the perceptions of their parents about the television ads.

**Methods:**

A cross-sectional study was conducted at schools in Punjab, India. Using multi-stage stratified random sampling, we included 722 school-going children aged 14–18 and their parents. A structured predefined questionnaire collected data using a four-point Likert scale. Descriptive statistics and binary logistic regression were used to draw our inferences.

**Results:**

About 46% of children were eating out > 3 times a week. Nearly 49% said they never looked at the expiry dates on the food packet, and 40% have yet to notice the quality certification. Nearly half do not understand the components of the food label, and 59% said they never changed their buying behavior because of the label. Only 37% of parents expressed their concerns about the timing of the ads when children watch television, while only 25.5% were concerned about the accuracy of the information. Concerns of the parents regarding the timing of the ads, and celebrity endorsements, were potential predictors for the increased frequency of eating out by the children.

**Conclusions:**

Low awareness regarding the utility of nutrition labels and minimal concerns of the parents increase the frequency of eating out. Unification of our existing policies regarding food labels and tv advertisements to develop family-centric interventions will bring us one step closer to improving the enabling environment to curb the growing menace of childhood obesity.

## Introduction

Poor diets contribute to the rising global burden of overweight/obesity and associated comorbidities, including cardiovascular disease, diabetes, and cancers [1]. Worldwide, excess weight is a common problem irrespective of the Sustainable Development Index status, resulting in 2.8 million deaths annually [2]. ﻿Rising rates of overweight and obesity are attributed to complex interactions between the societal, environmental, food industry, and individual factors [3]. Of these, diet-related factors are a substantial threat. While many people intend to adopt healthy behaviors, including better eating habits, most find implementation and sustainability a challenging task due to many distraction [4]. Of these, food packaging and television advertisements (tv ads) have significantly influenced the food choices made by people [5]. The Food Safety and Standards Regulations, 2018 define “Advertisement" as any audio or visual publicity, representation, or pronouncement made using any light, sound, smoke, gas, print, electronic media, internet, or website and includes through any notice, circular, label, wrapper, or other documents [6]. India has over 167 million households with television sets (out of 234 million), of which over 161 million have access to the Satellite TV [7].

Precisely, ﻿the tv ads have the potential to promote food and create positive marketing outcomes for both consumers and producers of these products [5]. They often support consumers in making healthy choices [8]. For instance, their role in increasing the frequency of handwashing with soap has been significantly better than other mass media advocacy interventions [9, 10]. Similarly, ads improved the iodized salts uptake and have been instrumental in reducing the prevalence of Iodine deficiency disorders in countries like Turkey and India [11]. The ads also generated considerable awareness against the adulteration of mustard oils by argemone that caused epidemic dropsy in India [12]. Further, the food labels have been crucial in generating awareness, followed by health advocacy campaigns against trans fats in food in developed countries [13, 14]. However, in countries like India with poor nutrition knowledge, Tv ads by the food industry are considered to be a primary deterrent to advocacy for healthy foods [15]. It has been reported that heavier media viewing is closely correlated with consuming more unhealthy food [16].

Likewise, food labels are acknowledged as the primary mode of communication between the food industry and the consumer, ultimately influencing their buying decision [17]. The information conveyed through food labels is considered to be cost-effective in generating nutrition-related awareness among consumers [14]. Therefore, standardized nutrition labels can effectively encourage the food industry to shift to a healthier ﻿menu [18]. However, most research on nutrition labels targets adult consumers, as it is considered that food labels do not alter a child’s preferences for buying a particular type of food [19, 20]. The food industry however tries to target and allure young consumers through colorful ads and popular cartoon characters, as they are highly susceptible to these marketing strategies [21]. The desire to buy food created by attractive food packaging is further aggravated through extensive advertisements even when the child is at home as there is a constant increase in children and adolescents screen time worldwide [22]. This opportunity is harnessed effectively by the food & beverage brands. They assign huge proportion of their budgets just for advertisements and brand promotions which is a rising cause of concern [23].

In addition to food labels and tv ads, parents significantly influence their children’s dietary behaviors and nutritional status. Evidence suggests that parental eating behaviors and their perceptions towards food labels, and tv ads are crucial in determining their children’s eating behavior [24, 25]. In response to the increasing concerns of consumers, industries use deceptive and inappropriate claims about the health impact of products termed ‘health washing’ [26]. This is done by re-framing the perceptions around unhealthy food and is supported through celebrities' endorsements, excessive campaigning, and using favorite cartoon characters [27]. Literature review suggests that better-aware consumers are less susceptible to health washing. Therefore it is pertinent to explore the current perceptions of children and their parents regarding food packaging and tv ads because these two factors significantly contribute to the rising burden of childhood obesity [28]. Most of the previous studies have targeted only the eating patterns of adolescents and have not considered their parental perceptions and concerns about tv ads that ultimately affect the eating-out frequency of their children [29–32]. Such recommendations will be crucial in developing family-centric interventions to curb the menace of childhood obesity. With this background, the present study aims to provide insight into the interpretation of food labels by school-going children to inform the stakeholders. The study's primary aim is to explore the noticeability of the food labels by the children, the information retrieved, and their role in modifying the eating out frequency. We also intend to study the effects of parental perceptions and concerns about television advertisements. Lastly, we attempt to explore the predictors of increased frequency of eating out of the children and relate it to their parent’s concerns about the tv ads.

## Methodology

### Study design and study period

We conducted a cross-sectional study between March-June 2022.

### Study settings

The study was conducted in a district from the Malwa region of Punjab, a northern state of India. With rapid economic development, India is now facing a double burden of malnutrition, due to the rising energy content of diets. The problem of childhood obesity is now steadily escalating [3]. The state of Punjab has a population of 2.7 crores, segregated into 22 districts. The state has a literacy rate of 76.7% and a sex ratio of 893, according to the 2011 census. Childhood malnutrition is a constant concern in the state of Punjab as the prevalence of undernutrition is still high, and the prevalence of overweight among children and adolescents is twice compared to the national average as per the reports of the Comprehensive National Nutrition Survey(2016–18) [33, 34]. This is closely linked to the high prevalence of overweight, obesity, and associated comorbidities like diabetes and hypertension among the adult population in Punjab. Higher prevalence translates to the rising burden of the determinants of overweight and obesity and may be attributed to the higher per-capita income of Punjab. Further, per the latest data compiled by the education department, Punjab has 28,568 schools, of which 67% (nearly 19,200) are state-run, and the remaining are in private (affiliated), unaided, associated, Adarsh (run on Public–Private partnership mode), and other categories.

### Specific study settings

The study was conducted in schools in and around Bathinda's district. The schools follow the guidelines issued by the Department of school education, transmitted through the District Education Officer. At the time of the study, there were 1017 schools, of which 346 were privately recognized and were a part of the Federation of Private Schools and Associations, Punjab [35].

### Study population

The study was conducted among school-going children aged 14–18 and their parents. The minimum age of 14 years was based on the fact that children may be able to activate the cognitive defenses required to withstand advertising effects after this age [36]. Parents could be of any age as long as they have children within the specified age range. Only one parent per child was invited to participate in the study.

### Sample size

A sample size of 706 children was calculated using the single population proportion formula after considering the change in behavior after looking at the food advertisements/labels to be around 15% [37], with a 95% confidence interval, a margin of error of 5%, design effect of 1.8, and a correction factor of 2 for urban–rural strata. We ultimately included a total of 722 children and their parents in the study. The sample size was calculated using an online statistical software “open-epi (Version 3.01)”.

### Sampling strategy

Data were collected using multi-stage stratified random sampling. In the first stage, we randomly select two blocks and two urban localities in Bathinda. The district education officer provided the list of schools in the urban and rural areas, and it was used as our sampling frame. In the next stage, we randomly selected five schools each from these four geographical areas after necessary permissions (*n* = 20). In urban areas, we selected three private schools vs. two government schools, while in rural areas, we followed a reverse pattern to have equal representation of government and private schools (*n* = 10 each).

In schools, all students were sensitized about the concepts of healthy eating and the importance of food labels during their morning assembly sessions by one of our co-authors. Respondents were sourced from a list of students provided by the schools. Stratified random sampling was used to achieve equal representation of children by gender, age, and the current class of education over the entire child sample. We randomly invited ten students (five boys and five girls) from the classes between the 8th and 12^th^. Students were then explicitly explained the purpose of the study. They were handed over a consent form and a study tool to be filled out by them and their parents. The study questionnaires were to be completed and returned to their class teachers within the next two days. Forms that were not filled in pairs or were incomplete were excluded from the analysis.

### Study tool

We used a structured predefined questionnaire typed and distributed among the study participants as per the protocol detailed subsequently. The study tool included two sections. Section A was meant for children and was having two parts. The first part of this section collected information about the socio-demographic details of the study participants, including name, father’s/gaurdian’s name, age, class, name of the school, per-capita monthly income, dietary preferences, the mean number of servings of fruits, vegetables, dairy products per day, salt (in grams) per month, preferences of eating out, behavioral practices and comorbidities in the family. The second part collected information sought by the participants from the food package using a structured questionnaire adapted from a previous study done in North India [38]. We collected data concerning the expiry date of food, its ingredients, vegetarian/non-vegetarian, nutritional information through food labels, quality certification, and price of the food packaging. Then specifically, we enquired about the data retrieved from the food labels and if the participants were affected by the information provided by these labels and changed their buying behavior in light of the information they interpreted.

Section B was meant for the parent of the children who participated in the survey. The initial part of the tool collected information about socio-demographic details and the prevalent behavioral practices in the family, like tobacco and alcohol consumption, comorbidities among family members in the house, and special dietary needs of the family members. The next part collected information regarding their perceived concerns about the tv ads for children's food. This information was collected using questions adapted from a previous Morley B et al. study, where the responses were captured through a four-point Likert scale [39].

### Validity of the tool

Initially, face-to-face interviews were conducted by the lead author with 20 school children and their parents attending a senior secondary school situated in the community outreach area of the department for acculturation of the initial draft of the tool. They were asked about their understanding of food labels and tv ads and any missing areas or concerns in the questionnaire. After each round of face-to-face interviews, the draft questionnaire was modified in consultation with three experts (authors of the present study). The experts suggested modifications to the three questions related to food labels, and one question from tv ads was reworded. The tool was sequentially translated into the Hindi and Punjabi language by a bilingual expert using a standard translation-back translation methodology [40]. Later, the bilingual questionnaire was shared with five community medicine faculty members who were not part of the study and were well-versed in Hindi and Punjabi Languages. The chosen experts assessed the tool for its content, structural construct, utility/ futility, arrangement, sections, domain-wise distribution of the questions, and face validity (overall sequence/ language and appropriateness). The experts explained the purpose of the study, followed by a Likert-type response sheet. Of the five faculty members approached, four experts reverted. The questionnaire was further modified as per the comments.

### Data analysis

Data were entered in Ms-excel and analyzed using SPSS version 21 after extensive data curation. The socio-demographic characteristics of the children from urban and rural areas were compared using a chi-square test and reported using p-values. ﻿Means and standard deviations of continuous variables were compared using an unpaired student t-test. Children's perceptions of food labels and parents' perceptions of TV ads were recorded on a 4-point Likert scale. We further explored the predictors of increased eating-out frequency and calculated the adjusted odds ratio with 95% CI (Confidence Intervals) using binary logistic regression analysis. The final model was constructed in three steps respecting a hierarchical approach for potential confounders. The variables were chosen after checking for multi-collinearity using the Variance Inflation Factor. The first model included the background characteristics of the children. In the next step, we included the variables related to the noticeability of food labels and the information retrieved from food labels. The final model was built using the significant variables with a *p*-value < 0.2 from the second model and parental concerns about their children's food habits. A p-value of less than 0.05 was considered significant.

### Ethical clearance

The study was approved by the institutional ethical committee of AIIMS Bathinda-wide letter number IEC/AIIMS/BTI/062, dated 19–02-2022. Informed consent forms were obtained from all the children and their parents before collecting the data for the study. Data was collected after discussing the purpose of the study using the participants’ information sheet. Participants were assured confidentiality, and data anonymity was maintained during the analysis. All methods were carried out in accordance with relevant guidelines and regulations.

## Results

We analyzed the results from the 722 child-parents duos. The proportion of the participants was comparable in the urban (*n* = 338, 46.8%) and rural areas (*n* = 384, 53.2%) (Table 1). There was a similar representation from each age group included in the study. The stratified representation of the student participants across gender, study class, dietary preferences, and frequency of eating out were similar in urban and rural areas. We observed non-significant differences in the mean consumption of the number of servings of fruits and vegetables per day, daily dairy product consumption, and salt intake per month. There were non-significant differences in the socio-demographic characteristics of the study participants, behavioral practices, and people living with comorbidities in the family except for per-capita monthly income, where a higher proportion of participants from urban areas reported higher per-capita income compared to rural areas. The most preferred food choices by the students included packaged snacks, followed by packed foods like ready-to-eat noodles, while carbonated drinks and salads were the least preferred. The preference when eating from the market was the same across urban and rural areas, except that a higher proportion of children from urban areas preferred proper meals than those from rural areas.

**Table 1:** Socio-demographic and eating characteristics of the study participants from the urban and rural areas

	**Urban areas**	**Rural areas**	**Total**	***P*-value**
	**N(Col %)**	**N(Col%)**	**N(Col %)**	
**Total**	338(46.8)	384(53.2)	722(100)	
**Completed age (Years)**				> 0.05
14	67(19.8)	80(20.8)	147(20.4)	
15	74(21.9)	64(16.7)	138(19.1)	
16	77(22.8)	93(24.2)	170(23.5)	
17	56(16.6)	72(18.8)	128(17.7)	
18	64(18.9)	75(19.5)	139(19.3)	
**Gender**				> 0.05
Male	186(55)	185(48.2)	371(51.4)	
Female	152(45)	199(51.8)	351(48.6)	
**Study class**				> 0.05
8	67(19.8)	80(20.8)	147(20.4)	
9	74(21.9)	64(16.7)	138(19.1)	
10	77(22.8)	93(24.2)	170(23.5)	
11	56(16.6)	72(18.8)	128(17.7)	
12	64(18.9)	75(19.5)	139(19.3)	
**Per-capita monthly income (**₹**)**				0.03
< 10,000	133(39.3)	182(47.4)	315(43.6)	
> 10,000	205(60.7)	202(52.6)	407(56.4)	
**Dietary Preferences**				> 0.05
Vegetarian	141(41.7)	155(40.4)	296(41)	
Non-vegetarian	104(30.8)	121(31.5)	225(31.2)	
Eggetarian	93(27.5)	108(28.1)	201(27.8)	
**Fruits and Vegetables servings/day (Mean ± SD)**	2.6 ± 1.7	2.4 ± 1.7	2.5 ± 1.7	> 0.05
**Dairy products servings/day (Mean ± SD)**	0.7 ± 0.5	0.7 ± 0.5	0.7 ± 0.5	> 0.05
**Salt intake per month (Mean ± SD)**	1.2 ± 0.7	1.2 ± 0.8	1.2 ± 0.7	> 0.05
**Prefer eating out**				> 0.05
Yes	158(46.7)	205 (53.4)	363 (50.3)	
No	180 (53.3)	179 (46.4)	359 (49.7)	
**Frequency of eating out**				> 0.05
< 1 time a week	80(23.7)	84(21.9)	164(22.7)	
2 to 3 times/week	101(29.9)	121(31.5)	222(30.7)	
> 3 times/week	157(46.4)	179(46.6)	336(46.5)	
**Preferred Food purchased**				
Fried food (Spiced Fritters)	155(45.9)	170(44.3)	325(45)	> 0.05
Salads	104(30.8)	103(26.8)	207(28.7)	> 0.05
Packaged snacks (chips)	206(60.9)	233(60.7)	439(60.8)	> 0.05
Sugar food (Rasgulla, barfi)	151(44.7)	146(38)	297(41.1)	> 0.05
Carbonated drinks (Coke)	87(25.7)	87(22.7)	174(24.1)	> 0.05
Packed food (Maggi, soups,)	197(58.3)	217(56.5)	414(57.3)	> 0.05
Proper meals for dinner lunch etc. (shahi paneer, butter naan)	195(57.7)	188(49)	383(53)	0.019
**Behavioural practices in family**				
Tobacco	10(3)	15(3.9)	25(3.5)	> 0.05
Alcohol	69(20.4)	81(21.1)	150(20.8)	> 0.05
**Comorbidities in family**				
Special dietary needs	37(10.9)	42(10.9)	79(10.9)	> 0.05
diabetes	82(24.3)	92(24)	174(24.1)	> 0.05
Hypertension	39(11.5)	53(13.8)	92(12.7)	> 0.05
Obesity	36(10.7)	53(13.8)	89(12.3)	> 0.05
Dyslipidaemia	3(0.9)	8(2.1)	11(1.5)	> 0.05
Cardio-vascular diseases	31(9.2)	28(7.3)	59(8.2)	> 0.05
Chronic Kidney Disease	7(2.1)	5(1.3)	12(1.7)	> 0.05
Cancer	1(0.3)	2(0.5)	3(0.4)	> 0.05
Anaemia/generalised weakness	57(16.9)	66(17.2)	123(17)	> 0.05
Others ailments	9(2.7)	7(1.8)	16(2.2)	> 0.05

Table 2 explores the information sought by the students from the food packaging. A larger proportion of the students (48.8%) said that they never looked at the expiry dates on the food packet, and a large number of students (46.0%) reported noticing the ingredients infrequently. Still, most students (71.7%) said that they always looked for vegetarian or non-vegetarian content in the food in case of confusion. Nearly 40% said they never noticed the food products order quality certification (40.2%). However, the price was noticed by the highest proportion of the study participants (90.6%) either regularly or infrequently. We also enquired about the information retrieved/interpreted only from the food nutrition label by the students. Most students did not find the food labels noticeable (61.9%) or readable (55.8%). More than half of them (52.2%) did not understand each component of the food label. In comparison, less than one-tenth (6.9%) could understand the nutritional information depicted on the label or interpret the meaning of the label (7.5%). Half of the students (49.3%) said they were never affected by those labels, and at least 59% said they never changed their buying behavior because of the label [41]. Of all the ingredients of the food, cholesterol was the most common food component (53.9%) that affected the buying behavior of the student, followed by proteins (47.1%) and fats (38.8%).

**Table 2:** Noticeability and utility of the food nutrition labels perceived by the children and their effect on the buying decisions

	**Always**	**Sometimes**	**Rarely**	**Never**
**Information sought from the food package**				
Expiry date	323 (44.7)	38 (5.3)	9 (1.2)	352 (48.8)
Ingredients	226 (31.2)	332 (46.0)	72 (10.0)	92 (12.7)
Veg./non-veg	518 (71.7)	99 (13.7)	24 (3.3)	81 (11.2)
Nutritional information	171 (23.7)	309 (42.8)	112 (15.5)	130 (18.0)
Food products order quality certification	169 (23.4)	133 (18.4)	130 (18.0)	290 (40.2)
Price	526 (72.9)	128 (17.7)	42 (5.8)	26 (3.6)
**Information retrieved from the food nutrition label on the package**				
Notice the label	44 (6.1)	20 (2.8)	211 (29.2)	447 (61.9)
readability of the label	40 (5.5)	41 (5.7)	238 (33.0)	403 (55.8)
Understand each component of label	35 (4.8)	38 (5.3)	272 (37.7)	377 (52.2)
Understand nutritional information depicted on label	50 (6.9)	50 (6.9)	305 (42.2)	317 (43.9)
Interpret the meaning of label	54 (7.5)	103 (14.3)	253 (35.0)	312 (43.2)
affected by the information on label	129 (17.9)	142 (19.7)	95 (13.2)	356 (49.3)
changed buying decision because of the label	67 (9.3)	103 (14.3)	128 (17.7)	424 (58.7)
**Ingredient affect your buying decision the most**				
Fat	280(38.8)	160(22.2)	175(24.2)	107(14.8)
cholesterol	389(53.9)	160(22.2)	77(10.7)	96(13.3)
Calories	250(34.6)	198(27.4)	176(24.4)	98(13.6)
Protein	340(47.1)	204(28.3)	100(13.9)	78(10.8)
Sugar	255(35.3)	277(38.4)	125(17.3)	65(9)
Minerals/Vitamins	177(24.5)	189(26.2)	164(22.7)	192(26.6)

Then, we explored the concerns of the parents of these students regarding food advertisement and how it affects the eating behavior of their children (Table 3). More than one-third of the parents expressed their deep concerns about the timing of the advertisements when children watch television (37.8%) and regarding the advertisements for food products that they perceived to be unhealthy at the time when their children are watching tv. Only one-fourth of the parents were concerned about the accuracy of the information regarding the advertised product's nutritional quality. The endorsement of unhealthy food products by a famous personality in the food advertisement bothered nearly 45% of the parents, and 34% did not like the idea of offering freebies with the food products. Almost 40% said that the number of advertisements about perceived unhealthy food aired during the prime-time should be evaluated.

**Table 3:** Concerns expressed by the parents about the pattern of food advertisements aired on television

	Not at all concerned	A little concerned	Somewhat concerned	Very concerned
**The extent of concerns about the:**				
**advertising of food products at times when children watch TV?**	80(11.1)	189(26.2)	180(24.9)	273(37.8)
**the advertising of UNHEALTHY food products at times when children watch TV?**	90(12.5)	148(20.5)	202(28)	282(39.1)
**TV food advertising provides accurate information about the nutritional quality of the product being advertised**	186(25.8)	158(21.9)	194(26.9)	184(25.5)
**the following aspects of food advertising at times children watch TV**				
The use of popular personalities or characters to promote unhealthy foods to children	121(16.8)	117(16.2)	156(21.6)	328(45.4)
Food advertising that promotes free toys or gifts with products	192(26.6)	155(21.5)	126(17.5)	249(34.5)
The amount of TV advertising of unhealthy food at times when children watch TV	135(18.7)	148(20.5)	150(20.8)	289(40)
TV food advertising that promotes only the healthy aspects of the product	190(26.3)	164(22.7)	117(16.2)	251(34.8)

We then assessed the predictors of increased frequency of eating out in the presence of noticeability of food labels and concerns shown by the parents regarding tv ads using a step-wise logistic regression (Table 4). It was seen that the increased frequency of eating out was not affected by the socio-demographic characteristics of the child. However, as per the final model, the frequency was affected in children who noticed ingredients, veg/non-veg status, and other nutritional information on the food labels, and among those who could interpret the meaning of the food labels, the given nutritional-related information on food labels. The eating out frequency of the children was significantly affected when the parents were concerned about the timings of airing the tv ads, unhealthy ads at the time when children usually watch tv, and famous personalities posing as brand ambassadors of the child's food.

**Table 4:** Logistic regression estimates to predict the effect of food labels and parent’s perceptions on eating out frequency among the study participants

Variables			**Model 1**	**Model 2**	**Model 3**
			aOR (95% CI)	aOR (95% CI)	aOR (95% CI)
Background Characters of the child	Gender	Female	1	-	-
		Male	1.18(0.88–1.58)	-	-
	Residence	Rural	1	-	-
		Urban	1.03(0.77–1.38)	-	-
	Per-capita Income (INR)	< 10,000	1	-	-
		> 10,000	1.11(0.82–1.5)	-	-
	Diet	Vegetarian	1	-	-
		Non-veg	0.95(0.67–1.35)	-	-
		Mixed	0.88(0.61–1.27)	-	-
Information sought from the Food labels	Expiry date	Always	-	1	1
		Sometimes	-	0.81(0.38–1.72)	0.68(0.31–1.49)
		Rarely	-	0.35(0.08–1.65)	0.42(0.09–1.99)
		Never	-	0.92(0.65–1.28)	0.94(0.66–1.34)
	Ingredients	Always	-	1	
		Sometimes	-	0.57(0.37–0.87)*	0.53(0.34–0.81) *
		Rarely	-	0.67(0.34–1.33)	0.63(0.33–1.21)
		Never	-	0.75(0.41–1.36)	0.65(0.35–1.21)
	veg/non veg	Always	-	1	
		Sometimes	-	1.92(1.18–3.11)*	1.77(1.09–2.87) *
		Rarely	-	0.8(0.31–2.01)	0.95(0.38–2.39)
		Never	-	1.49(0.83–2.67)	1.35(0.74–2.44)
	nutritional information	Always	-	1	
		Sometimes	-	1.37(0.87–2.14) *	1.5(1.2–2.34) *
		Rarely	-	1.81(0.99–3.3) *	1.98(1.1–3.55) *
		Never	-	0.96(0.56–1.63)	0.92(0.54–1.58)
	FSSAI	Always	-	1	-
		Sometimes	-	0.72(0.43–1.22)	-
		Rarely	-	0.94(0.54–1.63)	-
		Never	-	1.13(0.73–1.74)	-
	Price	Always	-	1	1
		Sometimes	-	0.93(0.6–1.45)	0.91(0.59–1.41)
		Rarely	-	1.75(0.85–3.61)	2.07(1.01–4.22)*
		Never	-	1.34(0.49–3.67)	1.49(1.1–4.06)*
	Language of the food label	Always	-	1	
		Sometimes	-	0.46(0.12–1.79)	-
		Rarely	-	1.03(0.35–3.07)	-
		Never	-	1.26(0.44–3.64)	-
	Readability of the food label	Always	-	1	
		Sometimes	-	0.94(0.25–3.52)	-
		Rarely	-	0.64(0.19–2.15)	-
		Never	-	0.45(0.13–1.55)	-
	Meaning of the food label	Always	-	1	1
		Sometimes	-	1.17(0.35–3.89)	0.45(0.2–0.99)*
		Rarely	-	1.74(0.59–5.15)	0.61(0.31–1.23)
		Never	-	1.52(0.5–4.61)	0.42(0.21–0.84)*
	Nutritional information available on the label	Always	-	1	1
		Sometimes	-	1.44(0.5–4.13)	1.5(0.96–2.34)*
		Rarely	-	1.39(0.56–3.46)	1.98(1.1–3.55)*
		Never	-	1.39(0.55–3.5)	0.92(0.54–1.58)
	Meaning of each term	Always	-	1	-
		Sometimes	-	0.42(0.17–1.17)	-
		Rarely	-	0.65(0.26–1.6)	-
		Never	-	0.47(0.09–1.14)	-
	Affected by the food label	Always	-	1	-
		Sometimes	-	0.82(0.47–1.44)	-
		Rarely	-	0.78(0.43–1.42)	-
		Never	-	0.88(0.55–1.4)	-
	Change buying decision due to label	Always	-	1	1
		Sometimes	-	1.71(0.85–3.46)	1.75(0.87–3.53)
		Rarely	-	1.46(0.73–2.93)	1.76(0.91–3.42)
		Never	-	1.23(0.67–2.25)	1.35(0.75–2.43)
Parental concerns about the tv ads	Timing of the ad	Not at all	-	-	1
		A little	-	-	0.16(0.08–0.31)*
		Somewhat	-	-	0.15(0.08–0.31)*
		Very	-	-	0.21(0.11–0.43)*
	Unhealthy ads at times when children watch TV	Not at all	-	-	1
		A little	-	-	0.72(0.37–1.38)
		Somewhat	-	-	0.45(0.23–0.85)*
		Very	-	-	0.83(0.43–1.61)
	TV ads not providing accurate information	Not at all	-	-	1
		A little	-	-	1.18(0.69–2.02)
		Somewhat	-	-	1.6(0.94–2.72)
		Very	-	-	1.69(1.2–2.99)
	Popular people as brand ambassadors	Not at all	-	-	1
		A little	-	-	0.45(0.22–0.93)*
		Somewhat	-	-	0.77(0.39–1.5)
		Very	-	-	0.5(0.28–0.9)*
	Alluring children with freebies	Not at all	-	-	1
		A little	-	-	1.4(0.8–2.44)
		Somewhat	-	-	1.18(0.61–2.26)
		Very	-	-	1.7(0.94–3.08)
	Amount of tv ads aired at prime time	Not at all	-	-	1
		A little	-	-	1.34(0.74–2.43)
		Somewhat	-	-	1.18(0.64–2.16)
		Very	-	-	0.86(0.47–1.57)
	Ads selective in focussing on healthy food part	Not at all	-	-	1
		A little	-	-	0.74(0.42–1.29)
		Somewhat	-	-	1.16(0.65–2.05)
		Very	-	-	0.93(0.56–1.54)

## Discussion

Overweight and obesity are a growing threat to children’s well-being globally, primarily driven by a highly susceptible obesogenic environment- a trap of unhealthy and highly processed foods worsened by a lack of physical activity and sedentary behavior. The escalation in prevalence is most rapid in low- and middle-income countries, including India. To counteract this, six main areas of government policymaking and programs are likely to hold the bulk of the authority. Food systems, including food labeling, are one of the thrust areas [42]. Within this context, this is among the few studies from India that assess the influence of food labels and parental concerns regarding food advertisements aired on television on children's eating-out behavior. Certain key findings are emerging from our study that can further our efforts toward effective food labels and advertisement policies in India. First, a very high proportion of children prefer eating out, with a higher preference for packaged food and minimal urban–rural disparities. Second, only one in four children sought nutrition information from the packaging, but only a few could effectively notice or read the food labels. Third, less than one-tenth of the children could understand the information and interpret the meaning of nutritional labels. Fourth, very few parents were concerned about how food ads are aired on television. Fifth, parental concerns significantly affected the eating-out frequency of their children.

More than three-fourths of our study participants preferred eating out at least > 2 times a week. This frequency is higher than previously reported estimates among similar age groups from India in the pre-pandemic period, depicting a rising eating-out trend [43]. A similar study from China has reported a lesser frequency of eating out [44]. With rapid economic developments, dietary patterns have changed, and eating out is the more acceptable culture in India [29–31]. Moreover, with the ease in the restrictions imposed during the COVID-19 pandemic, eating out has been even more frequent. Increased frequency of eating has been significantly associated with overweight and obesity in children from studies around the world [44–46]. The increased frequency can be attributed to the promotion of paid canteens in schools which refrain children from bringing their lunch boxes with them. We further observed that very few children sought nutrition information from the packaging, and even a smaller proportion could effectively notice or read the food labels. Previous studies have depicted low awareness among children regarding food nutrition labels and inferences drawn from them [17, 21]. ﻿Nutritional labels improve users' ability to identify healthy food judiciously [47–49]. However, children may not be familiar with the terms used in the labels, and thus the labels may not increase effectiveness [50–52]. Previous literature documents the lack of health perceptions of most children with nutrition labels is that their evaluations are based on previous pre-conceived perceptions of the product and label design as has been delivered through colorful advertisements and false health messages that have been endorsed by their celebrities, rather than relying on nutritional information [53]. Further, there is a need to improve the awareness among children regarding the nutritional information provided by the labels, and different interventions offer promising benefits [49]. The results also call for the implementation of easy-to-understand food labels tailored according to the knowledge base of the age groups targeted by the food material.

Previous studies have depicted better compliance and acceptability to better labels like Guideline daily amounts (GDA): Colored in black and white and showing the number of specific nutrients or calories per serving; traffic light system (TLS): Based on GDA, TLS adds traffic light colors (red, yellow, and green) to indicate nutrient levels, Apple label: Combining GDA and TLS features plus apple‑patterned outlines; ﻿ and Warning label: Black octagon with nutrients showing exceeding standard contents [21]. Within this context, it is pertinent to highlight a case study from Chile, where the Senate passed a landmark law mandating warning labels on the front of food packages containing excess fats, sugars, and salt. However, the initiative to implement the new law and its provisions faced significant resistance. Specific stakeholders were outspoken in discussing the threat of violating freedom of expression, international trade law and disregarding the principle of self-responsibility [54]. Despite being opposed vehemently, In June 2016, the first phase of the Chilean Food Labelling and Advertising Law and initial assessments reported the benefits of such legislation [55]. This case study highlights that implementing effective food labels may not be easy and will face strong resistance from the industrialist due to deep-rooted industry interference similar to what has been documented for the tobacco [56]. Hence, a large-scale advocacy drive is required in this regard involving different levels of stakeholders.

We observed only minor concerns of the parents regarding the unhealthy food ads aired on television, but their concerns significantly affected the frequency of eating out of children. Most parents were not at all concerned about the timing of these advertisements. A content analysis of the advertisements depicted that ﻿a higher proportion of food advertisements were seen on children’s channels than on youth channels, and the majority of the advertisements on children’s media promoted food that is high in fat, salt, sugar, and were advertised more on weekends than weekdays. ﻿Maximum advertisements were seen on weekdays between 8 pm–9 pm on children’s and youth channels, while on weekends, ﻿the prime time was 4 pm-5 pm for children’s media and 7 pm-8 pm for youth channels. The study concluded that advertisements targeted toward children and youth had specific timings [57]. Further, approximately half of the parents were okay with celebrity endorsement of food products. A previous study has depicted that consumers are most influenced in their food consumption behavior by the congruence between the celebrity endorsement and the recommended product and by the celebrity credibility [58]. At the consumer level, decreasing children's screen time will offer multiple benefits, with a reduced obsession with buying unhealthy food being one of them [59]. At the policy level, public health advertisements should compete with unhealthy advertisements for prime time slots through policy regulations that have depicted benefits in the long run [60].

There are a few strengths and limitations of this study that needs to acknowledge. Using a multi-stage stratified random sampling that accounts for urban–rural and age differences proves to be the biggest strength of this study, as this helps us to overcome our assumptions about the differences in dietary practices in urban and rural areas. Then, including the child-parent pair in our study gives us a better picture of the prevailing obesogenic perceptions among the study participants. However, we could not do a sub-group analysis based on the presence or absence of overweight and obesity in our study participants due to a lack of resources to estimate the BMI of the students. The presence of obesity can further the craving for sugary foods, which can affect buying behavior. Thus, the craving due to pre-existing overweight and obesity overruling the scientific plausibility cannot be ruled out. Further, we could not ascertain the noticeability of food labels by the parents and match it with the children’s responses. At last, the social-desirability bias may affect the response, and its effect on the reactions cannot be ignored.

Despite the limitations, our analysis has specific essential policy implications. Firstly, we need to emphasize the need to decrease the frequency of eating out by the children. For this, awareness regarding the harmful effects of refined packaged food among parents and teachers on the long-term health consequences for their children should be highlighted. Schools should be mandated to offer only healthy foods on their canteen menu [61, 62]. The government of India passed legislation prohibiting the sale of junk food in the vicinity of school campuses. Still, the directions are yet to reach the schools and must be vigorously enforced.

Further, we need to work on better food labels that are perceived and interpreted by the target clients in a better way. An influential nutritional label has various components that should be adhered to firmly. Lastly, we need to have a multi-centric approach at the family and community level to disengage the children and adolescents from the screens, which will also influence their buying behaviors. Lastly, we understand that some healthiest foods don’t have food labels, like fresh fruit and vegetables, nuts and fish, and government can endorse them as celebrities endorse jams and jellies. Future research can focus on the content and composition of food labels in the Indian context and compare them with developed countries. There is also a need to document the interference by the food industry that resists legislation regarding more useful food labels.

To conclude, our study demonstrates a high prevalence of preference for market food and low awareness concerning the nutritional value of the food products. There is an urgent need to reframe our policies regarding food labels. It will bring us one step closer to improving the enabling environment, including policies, regulatory frameworks, strategies, and accompanying monitoring and enforcement measures that will help curb the growing menace of childhood obesity.

## Data Availability

The data collected for the study are available from the corresponding author on reasonable request to protect the anonymity of the participants.
